# Collaborative Care and Everyday Planning in Care Counselling (CoPlan study): study protocol of a pragmatic randomized clinical trial and protocol changes to quasi-experimental evaluation during recruitment

**DOI:** 10.1186/s13063-026-10039-4

**Published:** 2026-11-03

**Authors:** Ingrid Hendlmeier, Martina Schäufele, Petra Meuer, Gisela Büchele, Ana Babac, Klaus Pfeiffer

**Affiliations:** 1 Department Social Work, Technical University of Applied Sciences, Mannheim, Germany; 2 Institute of Epidemiology and Medical Biometry, Ulm University, Ulm, Germany; 3 AOK Bavaria, Munich, Germany; 4 Department of Geriatric Rehabilitation, Robert Bosch Hospital, Stuttgart, Germany

**Keywords:** Long-term care, Intervention, Counselling method, Dyadic, Problem-solving, Quality of informal care, Randomized controlled trial, Feasibility

## Abstract

**Background:**

Family members are the primary source of support for older care recipients. Promoting cooperative strategies between family members may foster shared solutions to care-related challenges. Based on our previous research we developed a dyadic problem-solving intervention that equally addresses the needs of both caregivers and care recipients.

**Methods:**

This pragmatic randomized controlled clinical trial evaluates the implementation and effectiveness of the intervention under routine conditions within a national care counselling service setting. Due to recruitment obstacles and low participant enrolment the randomized design was modified to a non-randomized study. Main inclusion criterion for caregivers was a positive screening of desire to change the care situation, frequent caregiving challenges, or difficulty managing care. Participants are assigned to either the CoPlan intervention or control group with routine care counselling. Self-reported questionnaires are administered at baseline (*t*_0_) and 3 months post-intervention (*t*_2_) to collect endpoint data, with an intermediate non-endpoint assessment following the initial counselling (*t*_1_). Primary and secondary endpoint comparisons are based on data collected at *t*_2_ controlled for baseline values. Primary outcome is the quality of informal care (QiC), main secondary outcomes include caregiver self-efficacy, depressive symptoms, and positive aspects of caregiving. A process evaluation assesses intervention dose, fidelity, and experiences of interventionists, care counsellors of a German long-term care insurance, and participants.

**Discussion:**

This study represents one of the few translational studies in care counselling research. By promoting shared appraisals and open communication in a dyadic approach, the novel counselling method addresses a largely underexplored area. The difficulty in recruiting participants through care counsellors, along with the resulting study design modification, highlights the difficulties of conducting studies within real-world practice settings.

**Trial registration:**

Deutsches Register Klinischer Studien (German Clinical Trials Register), DRKS00032492. Registered on 5 September 2023 (current status: recruitment completed).

## Introduction

### Background and rationale {6a}

Informal caregiving is increasingly vital, yet care demand is surging due to population ageing and chronic disease while informal support capacity is shrinking [1]. Although caregiver interventions demonstrate efficacy, effect sizes are often modest [2] and implementation within formal care systems remains limited [3]. Research must therefore shift toward testing integrative caregiving models in real-world settings [1].

Communal coping in couples facing chronic illness enhances relationship quality and health outcomes [4]. In dementia dyads, psychological interventions show potential, though effectiveness across symptoms and burden varies [5]. Similarly, stroke research suggests caregiver-only interventions benefit caregivers, while dyadic approaches favour patient outcomes—though dyadic models can also yield caregiver benefits [6].

In Germany, 86% of long-term care recipients live at home, supported primarily by family or other informal caregivers [7]. To strengthen home-based care, care recipients and their informal caregivers have a statutory right to individual care counselling since 2009, according to § 7a of the German Social Code, Book XI (SGB XI). Care counselling aims to provide information, facilitate reflection on the care situation, and deliver targeted support through care planning and case management.

Evaluation reports [8, 9] show that the counselee, both caregivers and persons with need of care, generally view this service as helpful. However, these evaluations also highlight critical limitations: care counselling often lacks an evidence-based methodological framework, tends to be unstructured and incomplete (e.g. lacking implementation plans or case management evaluations), and 54% of counsellors report difficulties managing conflicts in informal care settings [9]. Although statutory standards require counsellors to hold a professional qualification in nursing, social work or social insurance, alongside 400 hours of mandatory training in care expertise, case management, and social law, only 40% of counsellors have received formal training in conflict resolution and problem-solving. Furthermore, counsellors report that delivering routine administrative and insurance information consumes substantial time, restricting opportunities for tailored, individualized support.

Previous efficacy [10] and effectiveness studies [11, 12] demonstrated that integrating problem-solving strategies [13], including a card-sorting assessment, into routine care counselling for distressed family caregivers is both feasible and effective in reducing caregiver depressive symptoms compared with standard care counselling [14, 15].

While our previous studies focused exclusively on highly burdened caregivers without the involvement of care recipients, the modified CoPlan approach aims to integrate the care recipients or at least their perspectives into counselling. Building on the same problem-solving steps, CoPlan places a greater emphasis on care-related everyday problems to foster balanced, mutually satisfactory solutions for both caregiver and care recipient. The intervention was developed in alignment with the existing German care counselling framework and national care counselling guidelines [16]. Reflecting this real-world setting, caregivers supporting individuals across a broad spectrum of chronic conditions are included.

### Choice of comparators {6b}

Participants in the control group receive usual care counselling. Consequently, participants in both groups may receive counselling within the same legal framework, with the aim of improving the primary and secondary outcomes.

### Objectives {7}

The aim of this pragmatic randomized controlled trial (RCT) is to evaluate the effectiveness of the CoPlan intervention against routine counselling. The primary endpoint is the quality of informal care, with main secondary endpoints comprising caregiver depressive symptoms, positive aspects of caregiving, resource utilization, self-efficacy, and caregiver burden. We hypothesize that the chosen dyad approach and the facilitation of balanced, realistic solutions will lead to better informal care quality, aligning with important policy goals. Furthermore, a process evaluation will be conducted to assess the intervention's implementation fidelity within a long-term care insurance setting, record participant feedback, and identify factors that facilitate or hinder implementation. This protocol is written according to the SPIRIT 2025 reporting guidelines [17].

### Trial design {8}

The CoPlan study was originally planned as a pragmatic randomized controlled trial (with parallel groups) conducted in cooperation with a long-term care insurance with recruitment period from 01/2024 to 10/2025. Due to recruitment difficulties, randomization was discontinued, and the study framework was changed from a superiority confirmation trial to a superiority exploratory trial aimed at exploring potential signals of superiority and treatment effects rather than providing regulatory confirmation.

## Methods: participants, interventions and outcomes

### Study setting {9}

CoPlan study is conducted within the care counselling regime of long-term care insurance in southern Germany. In the intervention group, counselling is conducted in person, either at the care recipient's home or at insurance service centres. In contrast, the control group has no specified requirements for the counselling setting, allowing for either face-to-face or telephone contacts.

### Eligibility criteria for participants {10}

Caregivers eligible for the trial have to provide care to a person who (a) is 18 years or older, (b) is categorized into a care degree (‘Pflegegrad’) from 1 to 5 indicating the degree of self-reliance restrictions according to the German Long-Term Care Act (‘Book XI of the Social Code’). Furthermore, participating caregivers have to (c) provide care and assistance in (instrumental) activities of daily living or supervision for ≥ 1.5 h per day on average or ≥ 10.5 h per week in total (including travel time), (d) be able to communicate in German, and (f) at least one of the following criteria should apply the following: (1) a pressing desire to change the current care situation, (2) frequent challenges in providing care, or (3) the current situation is often difficult for the caregiver to manage.

Caregivers are excluded when the person with need of care (a) does live in a nursing home, (b) will probably move into a nursing home within the next three months, (c) does receive palliative care, or (d) has a terminal illness or end-stage disease with life expectancy of less than six months. Further exclusion criteria referring to caregivers are (e) apparent cognitive impairment or a severe psychiatric diagnosis or (g) enrolled in another trial addressing caregiver issues or psychosocial outcomes.

### Eligibility criteria for interventionists

All counsellors in both groups hold a certified care counselling qualification according to the national legal requirements and have a prior healthcare sector education (such as social workers, nurses, or social insurance employees).

### Who will provide informed consent?

The study group of Technical University of Applied Sciences Mannheim (IH and PM) contacts caregivers by phone to outline the study’s objectives, phases of the study, and the procedure for obtaining consent form approval. All participants receive information leaflets and provide written consent for counselling and study participation as well as for data collection and processing. The care counsellors do the same for study participation and data collection. The ethics committee and data security officers from all institutions have approved the consent materials in advance.

### Explanation for the choice of comparators {6b}

The participant health insurance fund (AOK Bavaria) chose 15 districts for recruiting caregivers as potential study participants. Care counsellors (*n* = 31) were assigned as interventionists (*n* = 16) or control group counsellors (*n* = 15) by the insurance in each district, not randomly. Caregivers and persons with need of care allocated randomly to the control group receive the regular care counselling provided by the control group counsellor.

### Interventionists

The care counsellors of the long-term care insurance fund (AOK Bavaria) received an initial 2-day training in the CoPlan method, followed by a supervised 3-month period. The start of the recruitment was postponed for six months as a result of delayed approval of social data transfer by the nursing care insurance fund via the responsible ministry. The care counsellors are offered quarterly online group meetings to discuss experiences and questions about the application of the CoPlan method with the study team.

### Intervention description {11a}

The intervention is based on the problem-solving model developed by D’Zurilla and colleagues [13] and includes the following six problem-solving steps: (1) problem definition and facts, (2) optimism and orientation, (3) goal setting, (4) generation of alternatives, (5) decision-making, and (6) solution implementation and verification. The six components are viewed as a continuous and interlinking process rather than stages to be followed in serial order and are conducted within an existing counselling context. The approach is implemented in accordance with the German national guidelines for care counselling [16] and focuses on addressing daily issues and unmet needs of care recipients and their caregivers. Consistent with the typically low frequency of counselling sessions in routine care, the CoPlan intervention takes place in an initial personal contact in the flat of care recipient and at least two further follow-up contacts, usually conducted by phone.

The intervention was adapted from previous problem-solving programs for caregivers [10–12, 18] and co-developed with caregivers, care recipients, and counsellors through online surveys, focus groups, and pilot consultations. Assessment topics and session structure were modified from earlier programs to match the study's dyadic focus. A card-sorting task was implemented at the start of counselling to help identify problems and resources. The newly developed set of 14 theme cards (A7 card size) includes a header, three illustrative examples, and up to three icons on each card. The cards cover topics in home care such as indoor and outdoor mobility, communication, housing conditions, coping with chronic conditions, mood, care provision, health care and prevention, administrative tasks, simple pleasures in daily life, and wellbeing. Three additional cards with a different background colour focus on caregiver-specific topics: managing caregiving, wellbeing, and time for one’s own interests. One notable characteristic of the CoPlan counselling process lies in its concurrent and balanced attention to the needs and perspectives of both the care recipient and the primary caregiver. If the care recipient is willing and able to participate, the intervention is provided as dyadic counselling (CoPlan-Dyad); if not, it is offered to the caregiver alone (CoPlan-CarGiv).

The dyadic card sort procedure is carried out as follows: (A) The 14 cards are provided to the care recipient, who is instructed to assign each card to one of three categories (‘fine as it is’, ‘might need to change’, and ‘urgent need to change’). The 3-point scale is visualized with A7-sized cards arranged horizontally, resembling a traffic light. Urgent needs for change beyond those represented in the card set can be written on blank cards and allocated accordingly. (B) To clarify whether an urgent need for change is perceived by the care recipient, the caregiver, or both, the red traffic light is supplemented with two additional cards. This helps to distinguish between shared and individual needs for change. To see whether priorities are shared or distinct, the caregiver is first asked if he shares the care recipient’s view of the most pressing needs. In the next step the caregiver assigns the three designated caregiver cards and any additional labelled blank cards as needed. By working with the cards, the counsellor encourages the dyad to reflect on their present situation and to strengthen their confidence in managing challenges collaboratively. This may involve identifying functional care strategies, appreciating what already works well, normalizing certain stressors, and reinforcing the value of joint effort even in the face of differing urgent concerns. The counsellor contributes to the process by grouping related cards and by facilitating a balanced prioritization between individual and common objectives for change. During the process, the counsellor aims to acknowledge and integrate the potentially diverse viewpoints of the dyad, first promoting a constructive and respectful dialogue, second selecting one advice topic of caregiver and one of care recipient.

The selected advice topics are discussed in more depth, with facts being separated from assumptions. After gathering the relevant information, the counsellor supports the client or dyad in formulating a concrete and realistic goal to address the identified issue and to identify potential barriers to achieving this goal. Subsequently, the dyad is prompted to generate and document on a worksheet as many potential solutions to the problem as possible. Participants are reinforced for deferring evaluative judgments, and several techniques are suggested to enhance idea fluency (e.g. combining solutions, perspective-taking with a role model, or recalling prior solutions). Additional solutions are derived from the counsellor’s expertise. After compiling a thorough list of potential strategies, the interventionist supports the caregiver and care recipient in considering the potential effects of each approach and in individually judging its value and feasibility using the three categories: yes, uncertain, and no.

The last phase of the problem-solving process consists of implementing the chosen, carefully prepared individual care plan. In the next session with the counsellor, caregivers and care recipients provide feedback on how closely the results align with the anticipated and realized outcomes of their problem-solving attempts. This self-monitoring step is critical for understanding the factors influencing solution effectiveness and for informing the use of similar or alternative strategies in future problem situations.

During training, care counsellors are instructed to select the appropriate intervention format based on the care recipient’s span of attention and cognitive status. Depending on the care recipient’s ability to participate, the problem-solving intervention can be delivered either fully or partially in a dyadic setting (CoPlan-Dyad) or exclusively with the caregiver (CoPlan-CarGiv). In the CoPlan-Dyad format, counsellors tailor the care recipient’s involvement to their functional capacity. The card-sorting process begins from the care recipient’s perspective, allowing individuals with limited attention spans to engage initially, take a rest break, and re-engage during the subsequent goal-setting or solution-generation phases.

In the CoPlan-CarGiv format, the counsellors encourage caregivers to reflect on both their own perspective and that of the care recipient.

### Intervention: modification {11b}

Participants may discontinue the intervention and/or their study participation at any time by informing the care counsellors or study team, not returning questionnaires, or cancelling counselling appointments. Participants may continue to receive routine care counselling without any further study-related obligations.

### Strategies to improve adherence to intervention {11c}

During the intervention period, the counsellor conducted a minimum of two follow-up phone calls to monitor the implementation of solutions. In addition, all participants in the intervention group received nine printed information letters on caregiving (e.g. benefits of the long-term care insurance, relaxation, depression) by the study team. The description of the intervention is summarized in Table 1.

**Table 1:** Description of the CoPlan intervention (based on the TIDieR-checklist [19])

Item	Description
1. Brief name	Collaborative Care and Everyday Planning in Care Counselling (CoPlan study):
2. Why:	Problem-solving intervention that places a strong emphasis on care-related everyday problems aims to facilitate balanced solutions that are satisfactory for both the caregiver and the care recipient. Active involvement of the dyad or at least both perspectives is expected to improve quality of informal care.
3. What: materials	a) Recruiting schemes for care counsellors under regular care counselling conditions b) Worksheet ‘recruiting’ for pre-screening potential participants according the eligibility criteria c) CoPlan-card-sorting set with 17 theme cards and five heading cards for counselling assessment d) CoPlan-worksheet ‘goal setting and alternative solutions’ e) CoPlan-manual f) Nine information letters on caregiving
4. What: procedures	Cooperative care counselling by integrating perspectives of caregivers and care recipients • in card-sorting for analysis of actual situation with wishes for change and experienced burden • in problem solving with goal-setting and alternative solutions Counselling settings can be dyadic (caregiver + care recipient) (CoPlan-Dyad) or with caregiver only (CoPlan-CarGiv)
5. Who: provider	Licenced care counsellors from long-term care insurances with different professions (social workers, nurses, social insurance employees) and an additional qualification for care counselling according to the national legal requirements All counsellors were trained in a 2-day workshop by KP, IH and PM, and coached by KP and IH quarterly during the study in online group meetings
6. How: delivery	Individual, manual-based (short manual and materials are available from study group)
7. Where: location	In care-recipients home and ≥ 2 follow-up contacts by phone
8. When, how much: dosage	One initial home visit, ≥ 2 contacts by phone over 3 months The interventions were delivered within their usual working hours
9. Tailoring: what, how	• Using card-sorting method to identify relevant topics for care counselling (assessment) • Individual and communal goal setting • Care planning
10. How well: delivered as planned	Assessment of fidelity is based on analysis of: • card-sorting-assessments and working sheets ‘goal setting and alternative solutions’ • numbers and duration of contacts • study-related counselling documentations • protocols of online group-meetings and focus groups • questionnaires after the intervention period

### Intervention commitment care {11d}

Concurrent psychotherapy is an exclusion criterion due to its potential to confound the effects of the counselling intervention.

### Outcomes {12}

Primary and some secondary endpoints are self-assessed at three time points. Table 2 provides an overview of the primary and secondary outcomes and process evaluation data that are measured at the three time points. The primary endpoint is assessed at *t*_2_, 3 months after care counselling (*t*_1_).

**Table 2:** Timeline and content of CoPlan—assessments

**Timepoint**		**Caregiver**			**Care recipient**		
		***t***_**0**_	***t***_**1**_	***t***_**2**_	***t***_**0**_	***t***_**1**_	***t***_**2**_
Demographics	Gender, age, citizenship, civil partnership/marriage, housing situation, children, school-level-education, academic grades, advance directives	X			X^a^		
Care situation	Kinship, length and amount of caring, utilization of health service and counselling, resources (financial, knowledge) and social support in caregiving, utilization of offers of reconciliation of paid work and family care, care grade	X		X	X^a^		X^a^
Activities of daily living (10 items)	Barthel Index (BI)				X^a^		X^a^
Cognitive functioning (7 Items)	Extended Barthel Index (EBI) (6 items) and dementia (1 item)				X^a^		X^a^
Quality of informal home care	Own questions	rated by care counsellors after first session					
Primary outcome							
Quality of informal care (QiC) (27 Items)	QiC-Score: questions from Exemplary Care Scales (ECS), 12 items; Person-centred Practice Inventory-Staff (PCPI-S), 4 items; Dementia Management Scales (DMSS), 3 items; Person-centred care scale (PDC), 2 items; ReDiCare-study (harmful and abusive behaviour), 6 items	X		X			
Secondary outcome measures							
Participant reported quality of received care counselling und experiences with counsellor (33 items)	Own questions: Satisfaction (1 item), duration of counselling (1 item), plans for follow-up (1 item), received care counselling (13 items), experiences with counsellor (17 items); short-version for care recipient (19 items)		X	X		X	X
QiC subscale Caregiving competences and confidence (9 items)	QiC subscale I: questions from Exemplary Care Scales (ECS), 2 items; Dementia Management Scales (DMSS), 3 items; Person-centred Practice Inventory-Staff (PCPI-S), 4 items	X		X			
QiC subscale Person-centered caregiving behavoiur (8 items)	QiC subscale II: questions from Exemplary Care Scales (ECS), 6 items; Person-centred care scale (PDC), 2 items						
QiC subscale Inappropriate caregiving behaviour (10 items)	QiC subscale III: questions from Dementia Management Scales (DMSS), 4 items; ReDiCare-study (harmful and abusive behaviour), 6 items	X		X			
Resources related to wellbeing (5 items)	4 Items of PRUQ-CD Subscale “Utilization of Resources Related to Wellbeing” (URRWB) and 1 additional item	X		X			
Depressive symptoms (20 items)	CES-D	X		X			
Caregiver self-efficacy/sense of competence (16 items)	SCQ-AV: all 3 Subscales: “satisfaction with the care recipient”, “satisfaction with one’s own performance”, “consequences of involvement in care for the caregiver”	X		X			
Physical complaints—exhaustion (6 items)	GBB-24 Subscale exhaustion	X		X			
Consequences of caregiving on one’s own schedule (5 items)	CRA—modified subscale “impact on schedule” (4 items) and 1 additional item	X		X			
Lack of family support (3 items)	CRA—modified subscale “lack of family support”	X		X			
Satisfaction with received caring and with everyday life (9 items) by care recipient	Own questions (PQC-CR): received assistance, social interaction, daily structure, joyful activities, aids, housing, acceptance of disabilities, consciously accepted dependency, respect of individual wishes				X		X
Loneliness of care recipient (3 items)	UCLA-LS				X		X
Depressive symptoms and anxiety of care recipient (6 items)	PHQ-4 (4 items) and proxy-report (2 items)^a^				X X^a^		X X^a^
Other measures							
Desire to leave care (6 items)	DIT	X		X			
Positive aspects of caregiving (6 items)	Adapted version PAC – 6 items			X			
Quality of relationship (1 item)	Own question	X		X			
Perceived quality of care by caregiver (13 items)	PQC-CG: 8 ReDiCare-items (aids, nutrition, meeting the needs, treatments, mobility, appearance, satisfaction of care recipient with caring, medical assistance) and 5 new items (cooperation, depression, anxiety, sleep, and pain of care recipient)	X		X			
Enabling social participation (3 items)	Questions from Exemplary Care Scales (ECS) 2 items), Dementia Management Scales (DMSS) 1 item)	X		X			
Use of restraints	Own question	X		X			
Pain of care recipient (2 items)	Own questions (1 proxy-report + 1 self-report)				X X^a^		X X^a^
Sleeping disorder of care recipient (2 items)	Own items (1 proxy-report + 1 self-report)				X X^a^		X X^a^
Exhaustion of care recipient (1 item)	Own question				X		X
General life satisfaction (1 item)	OECD-Better Life Index	X		X	X		X
Counselling details							
Counselling setting and topics	Own questions	Written records by care counsellor					
Counselling costs							
Initial and following contacts till *t*_2_	Duration	Written records by care counsellor					

^a^Proxy-report by caregiver

*BI* Barthel Index [20], *CES-D* Centre for Epidemiological Studies – Depression Scale (CES-D) [21], *CRA* Caregiver Reaction Assessment [22], *DIT* Desire-to-Institutionalize Scale [23, 24], *EBI* Extended Barthel-Index [25], *ECS* Exemplary Care Scale [26, 27], *DMSS* Dementia Management Strategies Scale [28], *GBB-24* The Giessen Subjective Complaints List [29], *PCPI-S* Person-Centred Practice Inventory-Staff [30], *PDC* Person-Directed Care [31], *PHQ* Patient Health Questionnaire [32], *PAC* Positive Aspects of Caregiving [33], *PQC-CG* Perceived Quality of Care by Caregiver [12], *PQC-CR* Perceived Quality of Care by care recipient, *PRUQ-CD* Psychosocial Resource Utilization Questionnaire for Family Caregivers [34], *ReDiCare* Relieving Distress Caregivers [12], *SCQ-AV* Abbreviated Sense of Competence Questionnaire [35], UCLA Loneliness Scale [36]

*t*_0_ = before counselling, *t*_1_ = after counselling, *t*_2_ = 3 months after *t*_1_

### Primary outcome measure

The primary outcome, Quality of Informal Care (QiC), is operationalized as the total score of a 27-item scale encompassing exemplary and inadequate caregiving behaviours. Higher scores reflect superior quality of informal care. In line with prior research, the construct differentiates between two dimensions of caregiving quality: high-quality care (personalized, respectful, compensatory) and low-quality care (punitive, controlling, withdrawing) [27].

Items for assessing exemplary care were derived from different existing exemplary care scales: the Exemplary Care Scale (ECS) [26, 27], the Dementia Management Strategies Scale (DMSS) [28], the Person-Directed Care (PDC) [31], and the German Version of the Person-Centred Practice Inventory Staff (PCPI-S G-LTC) [30]. The items of exemplary care behaviour are rated on a rating scale of 0 ('never’) to 4 points (‘very often/always’). The inadequate care behaviour items were adapted from a questionnaire assessing harmful and abusive behaviours in a previous study [12], supplemented by some additional items from the DMSS [28]. The items of inadequate care behaviour are rated with 4 (‘never’) to 0 points (‘very often/always’).

The QIC assesses three dimensions: 'Caregiving competences and confidence' (9 items; Cronbach’s *α* = 0.78), 'Person-centered caregiving behaviour' (8 items; Cronbach’s *α* = 0.78), and‘Inappropriate caregiving behaviour’ (10 items; Cronbach’s *α* = 0.89). For the primary endpoint, these subscales were combined to a total score comprising 27 items (Cronbach’s *α* = 0.88).

### Secondary outcome measure

#### Participants reported quality of received care counselling

A 33-item questionnaire developed by the authors assesses the experiences of counselees (general satisfaction, experiences with counselling, perceived competence of counsellor) after the first counselling session and 3 months follow-up. This assessment enables a comparison between regular care counselling and the CoPlan method.

#### Utilization of psychosocial resources

One adapted subscale related to wellbeing (4 items) of the Psychosocial Resource Utilization Questionnaire for Family Caregivers of People with Dementia (PRUQ-CD) is used to measure the utilization of resources of caregivers [34]. Participants use a 5-point scale (1 = ‘never’, 5 = ‘very often’) to indicate the frequency with which they had utilized each resource (e.g. to do something for one’s own health and physical fitness) in the last 4 weeks.

#### Caregiver self-efficacy and burden

All three subscales (16 Items) of the abbreviated German version of the Sense of Competence Questionnaire (SCQ-AV) [35] are used in this study. Items are rated on a 5-point scale (1 = ‘agree’ to 5 = ‘disagree’).

#### Exhaustion

Subjective physical complaints are measured by the subscale ‘Exhaustion’ (6 items) of the Giessen Subjective Complaints List (GBB-24) [29], rated on a five-point scale, ranging from 0 (‘not existing’) to 4 (‘strong’).

#### Consequences of caregiving on one’s own schedule

Four items of subscale ‘impact on schedule’ of the Caregiver Reaction Assessment CRA [22] are measuring disruptions on time schedule, rated on a five-point scale, ranging from 0 (‘never’) to 4 (‘very often/always’).

#### Depressiveness

Depressive symptoms of informal caregivers are assessed by the 20-item Centre for Epidemiological Studies Depression scale (CES-D) [21, 37]. Total scores range from 0 to 60 with a score of ≥ 16 as an indicator of caregivers at risk for clinical depression.

#### General life satisfaction

Care givers and care recipients are asked to rate their general satisfaction with life on a scale from 0 to 10 [38].

#### Care recipients’ satisfaction with caring and everyday life

A self-developed nine-item questionnaire (Perceived quality of care and daily life by care recipient – PCQ-CR) examines the self-reported satisfaction with received care, social interaction, daily structure, joyful activities, aids, own mobility, respect of individual wishes, competence to accept disabilities, and consciously accepted dependency; five-point scale with 0 ‘never’ to 4 ‘very often/always’, higher scores show higher satisfaction.

#### Care recipients’ depressiveness and other complaints

Care recipient assesses own depression and anxiety with the 4 items of Patient Health Questionnaire (PHQ-4) [32], loneliness (UCLA 3-item loneliness scale [36]), and pain and sleeping disorders with own questions.

### Other measures

#### Caregiver characteristics

For caregivers, demographics, detailed conditions of the care situation, counselling use, and utilization of possibility of reconciliation of paid work and family care are assessed.

#### Care recipients’ characteristics, activities of daily living and cognitive functioning

Proxy-report by care giver is given for description of person with need of care: demographics, activities of daily living (measured with the Barthel Index (BI) [20]), higher cognitive functioning (comprehension, verbal expression, social interaction, problem-solving, memory/learning/orientation, and vision/neglect) with the Extended Barthel Index (EBI) [25], advance directives, and health and care service use.

#### Perceived quality of care by caregivers

The 13-item questionnaire PQC-CG measures different indicators of quality of care concerning the care recipient. Five new items (cooperation, depression, anxiety, sleep disorder, and pain of care recipient) had been added to the eight original PQC-CG questionnaire (aids, nutrition, meeting the needs, treatments, mobility, appearance, satisfaction of care recipient with caring, medical assistance) [12]. Items are rated on a 5-point scale from 0 (‘never’) to 4 (‘very often’).

#### Positive aspects of caregiving

Psychosocial benefits of caregiving among family caregivers are assessed with six items (items 2, 3, 4, 7, 8, 9) of the Positive Aspects of Caregiving (PAC) scale [33]. Items are scored on a 5-point scale from 1 (‘disagree a lot’) to 5 (‘agree a lot’).

#### Quality of relationship

The current and past quality of relationship is measured with one question (‘What is the quality of your current relationship with your relative?’), rated by care giver on a 5-point scale from 0 (‘very bad’) to 4 (‘very good’).

#### Family support

Measured with 3 items of subscale ‘Lack of family support’ of the Caregiver Reaction Assessment (CRA) [22, 39] on a five-point scale, ranging from 0 (‘never’) to 4 (‘very often/always’).

#### Desire to leave care

Care givers are asked to answer 6 questions concerning the likelihood of a moving of the care recipient into a nursing home [23, 24]. Scores range from 0 to 6 with higher scores indicating a greater desire.

#### Enabling social participation

Two items from the Exemplary Care Scale (ECS) [26, 27] and one item from the Dementia Management Strategies Scale (DMSS) [28] were chosen to get information if caregivers enable social activities and engagement of the care recipient.

#### Use of restraints

Care givers are asked about use of restraints or locking doors in last four weeks, rated with 4 (‘never’) to 0 points (‘very often/always’).

#### Process evaluation

In accordance with the Medical Research Council (MRC) framework for evaluating complex interventions [40] the major goals of the process evaluation are a) to examine how well the intervention was implemented as designed (focusing reach, dose, materials, and fidelity), b) to describe how caregivers and care recipients participated and responded to the counselling (‘participant responses’), and c) to explore and describe contextual factors that may influence the delivery of the intervention (‘context’ focusing on facilitating and hindering factors). *Dose of the counselling* is documented by standardized sheets by the counsellor (date, session duration, mode of contact, participants, content of counselling).

*Counsellors’ experiences* (both IG and CG) *and expectations concerning the CoPlan-counselling method* (only IG) are collected after training/before participant recruitment and after end of intervention period. Counselling self-efficacy (CSE) is examined with items of the Counsellor Activity Self-Efficacy Scale (CASES) [41, 42].

To assess *treatment fidelity in intervention group*, the CoPlan-counselling worksheets are rated by the study team, focusing on key intervention components like assessment of relevant counselling issues using a card-sorting method, integration of both perspectives in assessment, goal setting, solution finding, and monitoring phone calls.

Additional qualitative data will be extracted from focus group meetings with interventionists asking for feedback on their experience with the intervention approach (materials, feasibility, etc.) after the initial training, in middle and at end of the intervention period. Phone-based interview with selected care givers of IG supplements the quantitative data of care giver experiences.

### Participant timeline {13}

After recruitment by care counsellors, enrolment is continued by study group to randomize participants to intervention or control group and to get written informed consent and first assessment (*t*_0_). Counselling in intervention group and control group is arranged by care counsellor. A second assessment is sent out 7 days after counselling (*t*_1_) for evaluating the counselling. The intervention group receives two follow-up contacts by their counsellor within intervention period of 3 months. Prior sending out last assessment (*t*_2_) 30 days after counselling all participants receive a phone call by study group. A participant timeline see Table 3.

**Table 3:** CoPlan—participant timeline © 2025 Chan

	Trial period					
	Enrollment		Post-randomization			
**Timepoint**^**a**^	−*t*_i_ to 0	0	*t*_0_	Care counselling	*t*_1_	*t*_2_ and close out
					(7 days after care counselling)	(90 days after counselling)
**Enrolment:**						
Eligibility screen by care counsellors	X					
Transfer of caregivers’ contact data to study team via encrypted email	X					
Eligibility screen by study team	X					
Oral informed consent	X					
Randomization		X				
Written informed consent			X			
**Intervention/comparator:**						
CoPlan care counselling^b^				X		X
Care counselling as usual				X		X
**Assessments**: Details see Table 2			X		X	X

^a^List target timepoints and acceptable time windows in this row (+ 30 days)

^b^Continuous delivery of intervention via telephone monitoring

### Sample size {14}

The sample size calculation with GPower 3.1.9.4 [43] is based on a two-sided *t*-test (*α* = 0.05) comparing groups at the 3-month follow-up (*t*_2_), an assumed moderate intervention effect compared to the control condition (Cohen’s *d* = 0.40), a test power of 1−*β* = 0.80, and a control-to-intervention ratio of 1.00. A preliminary sample size of *n* = 200 was initially determined. For the final ANCOVA analysis, this size was adjusted using a design factor [44] based on an anticipated baseline-to-follow-up correlation of *ρ* = 0.70 (derived from our previous studies). The final required sample size was calculated as 200 × (1—0.70^2^) = 102 participants. Accounting for an anticipated 20% drop-out rate between *t*_0_ and *t*_2_, also based on data from our previous studies, the final recruitment target was set at 128 participants.

### Recruitment {15}

The study participants are recruited by the care counsellors from a long-term care insurance provider (AOK Bavaria). Three distinct recruitment strategies were applied to access the target group: (I) enrolment during routine care counselling initiated by the caregiver’s or care recipient’s request, and (II) proactive enrolment during mandatory counselling visits (according to § 37.3 of the German Social Code, Book XI) or (III) counselling following a first classification or later reclassification into a care level. The recruiting strategies are coordinated, evaluated, and if necessary, modified with the administrative officials within the health insurance provider.

The recruiting care counsellors of the insurance inform caregivers about the study, administer a short pre-screening to identify potentially study participants, and ask for consent to forward the caregiver contact details to the study team of the Technical University of Applied Science, Mannheim. A member of the study team informs about further details of the study (via phone and written information) to enable the caregiver to make an informed decision whether to participate in the study or not. The signed consent form must be sent in a prepaid reply envelope to the study team of the Technical University of Applied Science, Mannheim. Each caregiver who requests care counselling and agrees to participate in the study is randomized to either the intervention or control group and consequently receives counselling from a counsellor trained in the CoPlan method or from a counsellor not trained in the method.

### Protocol change concerning recruitment and study design {15} {8}

Prior to study registration and participant recruitment, the study design was modified from cluster randomization to individual randomization at the request of the health care insurance and with approval from the funder. However, individual randomization by assigning the participants to the specific counsellor post-randomization introduced logistical complexities. Specifically, caregivers could not receive immediate counselling upon baseline assessment, and there was a potential risk of having to switch from the recruiting counsellor to another counsellor.

All recruitment barriers and reasons for under-recruitment will be evaluated in a formal process evaluation. By November 2024, the recruitment numbers were substantially lower than projected (*n* = 41 of planned *n* = 128; 32%), Consequently, to reach the target sample size, randomization was discontinued on December 15, 2024, and recruitment transitioned to a quasi-experimental design with employing two key strategies: (1) routine recruitment by care advisors in both trial arms, and additional (2) proactive telephone outreach to family caregivers using computer-generated call lists administered by care advisors and long-term care insurance staff outside the trial arms. Participants recruited via telephone outreach were allocated to either the control or intervention group based on the care recipient’s residential postal code, which determines the responsible regional care advisor.

To gather additional data on intervention feasibility, four counsellors from another long-term care insurance provider (Social Insurance for Agriculture, Forestry and Horticulture) were trained in May and June 2025 and evaluated from July to October 2025 using a streamlined assessment protocol with a single anonymous *t*_1_ assessment. Conducting further non-anonymous assessments was not feasible within the available timeframe due to additional data security consent requirements mandated by the responsible federal authority.

## Assignment of interventions

### Allocation: sequence generation {16a}

To minimize delays in consultation, participants are consecutively randomized in a 1:1 allocation ratio immediately following the provision of informal oral consent in this low-risk study (final participation depends on postal return of the signed consent form and *t*_0_ assessment). Group allocation is based on stratified block randomization with variable block sizes, generated by Ulm University using the ROM randomization software. Stratification was conducted according to the care recipient’s cognitive status (absent vs. present manifest cognitive impairment), defined via the Cooper Scale: absent (no/mild impairment: Cooper Scale score ≤ 2) versus present (moderate/severe impairment: Cooper Scale score > 2) [45].

### Allocation: concealment mechanism {16b}

The randomization lists are concealed stored in the backend in a database installed locally in the study centre (Technical University of Applied Sciences Mannheim).

### Allocation: implementation {16c}

Members of the study centre have access by entering their initials, the screening number and the stratum information in an input mask (front end). In response, the program outputs the group allocation and the random ID.

For an informed consent before enrolment, participants are informed about the possible study arms with different counsellors, acknowledging that the initial care counsellor providing initial information may not be the subsequent consultant.

### Blinding {17a}

Counsellors are not blinded as they provide in the intervention group the CoPlan-counselling and in control group the care counselling as usual. Participants are blinded but may have been able to discern their group allocation as the distinct nature of the new intervention (card-sorting, worksheets) compared to their possible prior experience with care counselling is obvious. Participants should complete questionnaires themselves, so study group members are not engaged in outcome-assessment.

### Blinding: emergency unblinding {17b}

N/a. Care counsellors are unblinded; participants of intervention and control group get care counselling according German Social Code Book XI (SGB XI).

## Data Collection, management, analysis, and monitoring

### Data collection methods {18a} {18b}

Primary and some secondary endpoints are self-assessed at baseline (*t*_0_) and at *t*_2_ (3 months after intervention) by caregivers and if possible, by persons in need of care, in written questionnaires. Some secondary outcomes and data for process evaluation are collected in an additional assessment immediately after the counselling (*t*_1_). Table 2 provides an overview of the primary and secondary outcomes and process evaluation data that are measured at the three time points.

The study group inform the caregiver about the assessment points during the recruitment phone call. Subsequently, printed questionnaires with individual code numbers are mailed to the caregivers for completion. Data on the care recipient's perspective were collected via a separate, self-administered questionnaire, where feasible. A subsequent telephone contact is conducted to review the questionnaire for completeness and to clarify any ambiguities. Caregivers are then requested to return the questionnaires using the enclosed return envelope. Prior to sending the final assessment, the study group contacts the caregiver in a further telephone call. Telephone reminders and reviews are used to enhance survey participation and minimize attrition and missing data.

Process evaluation data are derived from assessments of the counselling, which are completed by caregivers and care counsellors.

### Data management {19} {27}

Original questionnaires are scanned, classified, recognized, verified by the study team at the Technical University of Applied Science Mannheim and transferred with the TeleForm®-software (version 10.8, Electric Paper Informationssysteme GmbH, Lüneburg, Germany) into the database of the data centre of the Ulm University. All data communication processes, data storage, and data encoding are specified in detail in a data protection contract according to the European General Data Protection Regulation. Data preparation and main outcome data analysis will be performed at Ulm University with R [46] and SAS software [47]; data preparation and further data analysis will be performed at Technical University of Applied Science Mannheim and Robert Bosch Hospital with SPSS [48] and R [46].

### Statistical methods {20a} {20b} {20c}

Descriptive analyses will be carried out to baseline characteristics of both groups. Group differences of the primary and secondary endpoints will be tested on a two-sided significance level of 5% with analyses of covariance (ANCOVA).

Group differences at baseline (*t*_0_) and other comparisons will be analysed according to the variable type with *t*-test for normally distributed continuous variables, Mann–Whitney *U* test for ordinal or not normally distributed continuous variables, and *χ*^2^-test or Fisher exact test for categorical variables.

For the planned intention-to-treat (ITT) analysis, missing values for withdrawn subjects or participants with missing data at *t*_1_ to *t*_2_ will be considered by an adequate statistical method (e.g. Maximum Likelihood Multiple Imputation). Following the discontinuation of randomization, the statistical analysis plan was revised from a standard RCT framework to a quasi-experimental evaluation design to address potential selection bias, baseline imbalances, and data clustering. Baseline characteristics will be systematically compared between study arms. Confounding and selection bias will be mitigated using propensity score methods (e.g. matching or inverse probability weighting) combined with multivariable covariate adjustment in the primary ANCOVA models. To account for counsellor and district effects, multilevel linear mixed-effects models will be fitted, nesting individual participants (Level 1) within counsellors/districts (Level 2). If sample sizes permit, participants recruited prior to and following the design change will be compared. If subgroup sizes are sufficient, recruitment cohort (pre- vs. post-amendment) will be integrated as a subgroup factor and interaction term (Group × Cohort) to evaluate design-induced shifts in outcome measures. Should sample sizes prove inadequate for these analyses, this limitation will be explicitly acknowledged and discussed.

### Monitoring {21a} {21b} {23}

As no funding was allocated by the sponsor for an external data monitoring committee or external audits, data quality and integrity were routinely monitored and assured by Ulm University. No interim analyses are planned.

### Harms {22}

Although adverse events of the low-risk intervention or assessment procedures are not expected, the study team explores any serious events since *t*_0_ in the final phone call before sending *t*_2_-questionnaire.

## Ethics and dissemination

### Ethics approval {24} {25}

Ethical approval was obtained from the Ethics Committee of the University of Ulm, Germany (committee’s reference number: 154/23). Data security officers of the participating long-term careinsurances (AOK Bavaria), the Technical University of Applied Sciences Mannheim, the University of Ulm, as well as the Bavarian State Ministry of Health and Care agreed to the datamanagement and security protocol as well as to the participant information and consent form. All protocol modifications are communicated with the ethics committee, and trial registry.Modifications in the data management and security protocol are also communicated with the involved institutions.

### Consent to participate {26a}

The study group of Technical University of Applied Sciences Mannheim (IH and PM) informs the caregiver and care recipient about study’s objectives by phone. For obtaining written informedconsents the consent forms are sent by post to potential study participants. Returned written consent forms are stored by study group of Technical University of Applied Sciences Mannheim (IH ofPM) and the care counsellor in charge is informed via secure email communication about study participation.

### Consent or assent: ancillary studies {26b}

n/a; no ancillary studies are planned.

### Ancillary and post-trial care {30}

Not applicable. Ancillary studies are not planned and harms from trial participation are not to be expected. All participants will get information about post-trial care by study team in the last phone call before *t*_2_ about the possibility to get further counselling by care counsellors according to Social Code Book XI (SGB XI).

### Dissemination {31a, b, c}

The study materials and findings will be published in German as part of a project report available on the website of the National Association of Statutory Health Insurance Funds (www.gkv-spitzenverband.de). Study results in English will be published in scientific journals. Authorship follows eligibility guidelines, and professional writers are not planned for use.

## Discussion

This study aims to evaluate the effectiveness of a new counselling method delivered by care counsellors within long-term care insurance services, representing one of the very few translational studies in care counselling research. By developing this problem-solving intervention, we address the important role of shared appraisals in facilitating effective dyadic coping among individuals managing chronic illness [49], emphasizing the necessity of open communication within caregiver–care recipient dyads. In cases where the care recipient is unable to participate, their perspective is nonetheless integrated. This approach is expected to improve informal caregiving quality among individuals facing caregiving challenges, a relatively under-researched domain [50]. Although illness-specific interventions allow for better tailoring to the needs of caregivers and care recipients, they introduce operational complexity into routine practice environments that provide counselling across multiple illness groups. Insights from our previous translational studies [11] [12], alongside typical one-day counsellor training workshops and the standard delivery format of two to three sessions, indicate that only a moderate level of intervention complexity is feasible for this real-world setting. As the intervention was adapted from our previously tested and implemented problem-solving program [11, 12], we evaluated the CoPlan intervention directly within an effectiveness trial. Involving counsellors as well as caregivers and care recipients during the development phase was intended to enhance its feasibility and acceptability. Furthermore, the intervention materials were adapted for use in the insurance company's online platform and are evaluated in a small feasibility study outside the scope of this protocol. At the request from the long-term care insurance provider during the planning phase, the trial design was modified from cluster to individual randomization. Although this change reduced the required sample size, assigning counsellors to study arms post-randomization introduced logistical complexity into the recruitment process. Because recruitment was conducted by care counsellors who faced time constraints and were unfamiliar with the methodology and value of randomized trials, enrolment fell far short of projections. By discontinuing randomization and providing additional personnel for recruitment, the study was able to proceed within an exploratory framework. Caregivers frequently experienced high caregiver burden and feel not able to meet the study requirements. Concurrently, care counsellors tended to preselect against caregivers with high perceived burden, deeming them unsuitable for trial participation. In contrast to our previous studies, the funder did not support an individual monthly coaching component in this study, which had previously served as a key driver of counsellor engagement. Instead, counsellors' participation was largely driven by directives from superiors, introducing a potential confounder. Furthermore, recruitment was delayed by 6 months following staff training, primarily due to a lengthy and complex approval process for social data transfer involving the long-term care insurance provider and the responsible ministry. Alongside individual-level factors such as high workload, these challenges likely compromised motivation across all study procedures, particularly participant recruitment. Additionally, the complex decision-making hierarchy within the health insurance organization posed a structural barrier to implementation. All these implementation hurdles offer valuable insights into the lower-than-projected recruitment figures and highlight the necessity of adjusting the study design and objectives. Several limitations of this study should be noted. First, care counsellors’ awareness that their practice is being evaluated and compared may introduce bias in participant recruitment and the fidelity of the counselling delivery in both groups. Second, in this pragmatic trial, outcome data are collected via self-administered paper questionnaires completed directly by caregivers and care recipients, rather than through structured evaluations conducted by trained assessors. This reliance on self-reported measures, coupled with potential item ambiguity for respondents, may increase the rate of missing data.

Finally, relying on caregiver self-assessment to measure quality of informal care as primary outcome carries a risk of social desirability bias, as caregivers may report improvements even if their actual caregiving practices remain unchanged. However, because participants in both the intervention and control groups received care counselling (either the CoPlan approach or standard counselling as usual), both arms are exposed to professional support. Consequently, any observed between-group differences in the primary outcome can be more confidently attributed to the specific components of the intervention rather than general counselling or attention effects.

Despite these limitations and the recruitment barriers encountered, evaluating the intervention within routine care settings remains essential for assessing its effectiveness, identifying key factors for successful training and implementation, and establishing its real-world feasibility. The findings from the CoPlan study are expected to strengthen the evidence base for dyadic caregiver interventions, thereby supporting efforts to enhance the quality of informal care.

## Trial status

This is version 3 from 2026/09/25. The enrolment of participants started in January 2024 and continued until 31 October 2025. At time of the first submission of the study protocol (2025/30/31) the recruitment was completed and the last participant out was on 2025/10/31. Because recruiting participants in this routine setting was more challenging than expected, we had to revise both our recruitment strategy and the overall study design, including suspending randomization (December 2024). These changes and several extensions of the recruitment and evaluation period (the most recent on 2025/09/30), led us to submit the study protocol later than planned.

## Data Availability

Study and training materials for the CoPlan intervention will be provided in German by the study team (KP, IH) upon reasonable request. De-identified participant data are available from IH on reasonable request. This study protocol does not contain any data or results of the evaluation. Scientific trial members of the Technical University of Applied Sciences Mannheim, the Robert Bosch Hospital, and the study statistician from the University of Ulm will have access to the final data set. To ensure confidentiality, data distributed to members of the study team will be blinded of any identifying participant information.

## References

[1] Schulz R, Beach SR, Czaja SJ, Martire LM, Monin JK. Family Caregiving for Older Adults. Annu Rev Psychol. 2020;71(1):635–59. 10.1146/annurev-psych-010419-050754 PMC7291827 31905111

[2] Walter E, Pinquart M. How effective are dementia caregiver interventions? An updated comprehensive meta-analysis. Gerontologist. 2020;60(8):e609–19. 10.1093/geront/gnz118. 33226434

[3] Gitlin LN, Marx K, Stanley IH, Hodgson N. Translating evidence-based dementia caregiving interventions into practice: state-of-the-science and next steps. Gerontologist. 2015;55(2):210–26. 10.1093/geront/gnu123. PMC4542834 26035597

[4] Rentscher KE. Communal coping in couples with health problems. Front Psychol. 2019;10:398. 10.3389/fpsyg.2019.00398. PMC6414458 30894824

[5] Poon E. A systematic review and meta-analysis of dyadic psychological interventions for BPSD, quality of life and/or caregiver burden in dementia or MCI. Clin Gerontol. 2022;45(4):777–97. 10.1080/07317115.2019.1694117. 31752633

[6] Bakas T, McCarthy MJ, Miller EL. Systematic review of the evidence for stroke family caregiver and dyad interventions. Stroke. 2022;53(6):2093–102. 10.1161/STROKEAHA.121.034090. PMC9133104 35264010

[7] Federal Statistical Office of Germany. People in need of long-term care, by type of care. 2024. https://www.destatis.de/EN/Themes/Society-Environment/Health/_Graphic/_Interactive/long-term-care-type.html. Accessed 01 Sep 2025.

[8] Wolff JK, Pflug C, Rellecke J, Rieckhoff S, Dehl T, Nolting HD. Evaluation der Pflegeberatung und Pflegeberatungsstrukturen gemäß § 7a Absatz 9 SGB XI. [Evaluation of care counselling and care counselling structures according §7a section 9 social care act XI] Berlin: IGES Institut; 2020. www.gkv-spitzenverband.de/media/dokumente/pflegeversicherung/beratung_und_betreuung/pflegeberatung/20200331_IGES_Evaluation_Pflegeberatung_Abschlussbericht.pdf. Accessed 1 Sep 2025.

[9] Wolff JK, Pflug C, Meier D, Roll P, Dehl T, Topalov L, et al. Evaluation der Pflegeberatung und der Pflegeberatungsstrukturen gemäß der gesetzlichen Berichtspflicht nach § 7a Abs. 9 SGB XI [Internet]. Berlin: IGES Institut; 2023 [cited 2025 Sep 1] p. 412. https://www.gkv-spitzenverband.de/pflegeversicherung/forschung/modellprojekte/pflege_abgeschlossene_projekte_8/eva.jsp. Accessed 1 Sep 2025.

[10] Pfeiffer K, Beische D, Hautzinger M, Berry JW, Wengert J, Hoffrichter R, et al. Telephone-based problem-solving intervention for family caregivers of stroke survivors: a randomized controlled trial. J Consult Clin Psychol. 2014;82(4):628–43. 10.1037/a0036987. 24911421

[11] Pfeiffer K, Hautzinger M, Patak M, Grünwald J, Becker C, Albrecht D. Problem-solving in caregiver-counselling (PLiP study): study protocol of a cluster randomized pragmatic trial. BMC Geriatr. 2017;17(1):64. 10.1186/s12877-017-0456-x. PMC5339956 28264663

[12] Pfeiffer K, Theurer C, Büchele G, Babac A, Dick H, Wilz G, et al. Relieving distressed caregivers (ReDiCare study): study protocol of a randomized pragmatic trial. BMC Geriatr. 2021;21(1):4. 10.1186/s12877-020-01941-w. PMC7787127 33407195

[13] D’Zurilla TJ, Nezu AM, Maydeu-Olivares A. Social problem solving: theory and assessment. In: Chang E, D’Zurilla TJ, Sanna LJ, editors. Social problem solving: theory, research, and training. Washington: American Psychological Association; 2004. p. 11–27.

[14] Pfeiffer K, Hautzinger M, Becker C, Albrecht D, Pendergrass A, Leonhardt R, et al. Problemlösen in der Pflegeberatung - ein Ansatz zur Stärkung der Pflegeberatung nach § 7a SGB XI. Hürth: CW Haarfeld GmbH; 2018. (GKV Schriftenreihe Modellprogramm zur Weiterentwicklung der Pflegeversicherung; vol. 14).

[15] Töpfer NF, Pfeiffer K, Wrede N, Büchele G, Wilz G. Effectiveness of stepped counseling on depression in distressed family caregivers: A randomized pragmatic trial (ReDiCare). J Consult Clin Psychol. 2026;94(5):293–304. 10.1037/ccp0001004. 42241064

[16] National Association of Statutory Health Insurance Funds [GKV-Spitzenverband] (editor). Richtlinien des GKV-Spitzenverbandes zur einheitlichen Durchführung der Pflegeberatung nach § 7a SGB XI vom 7. Mai 2018 (Pflegeberatungs-Richtlinien) geändert durch Beschluss vom 09.01.2024. 2024. https://www.gkv-spitzenverband.de/media/dokumente/pflegeversicherung/beratung_und_betreuung/pflegeberatung/2024-01-09_Pflegeberatungs-Richtlinien.pdf. Accessed 1 Sep 2025.

[17] Chan AW, Boutron I, Hopewell S, Moher D, Schulz KF, Collins GS, et al. SPIRIT 2025 statement: updated guideline for protocols of randomized trials. Nat Med. 2025;31(6):1784–92. 10.1038/s41591-025-03668-w. 40295741

[18] Dautel A, Eckert T, Gross M, Hauer K, Schäufele M, Lacroix A, et al. Multifactorial intervention for hip and pelvic fracture patients with mild to moderate cognitive impairment: study protocol of a dual-centre randomised controlled trial (OF-CARE). BMC Geriatr. 2019;19(1):125. doi/10.1186/s12877-019-1133-z. PMC6492424 31039754

[19] Hoffmann T, Glasziou P, Boutron I, Milne R, Perera R, Moher D, et al. Better reporting of interventions: template for intervention description and replication (TIDieR) checklist and guide. BMJ. 2014;348:g1687. 10.1136/bmj.g1687 24609605

[20] Mahoney FI, Barthel DW. Functional Evaluation: The Barthel Index. Md State Med J. 1965;14:56–61. 14258950

[21] Radloff LS. The CES-D Scale: A Self-Report Depression Scale for Research in the General Population. Appl Psychol Measure. 1977;1(3):385–401. 10.1177/014662167700100306

[22] Weyer-Wendl H, Tamm M, Walter P. Evaluierung der deutschen Version des Caregiver Reaction Assessment-Fragebogens bei betreuenden Angehörigen von Patienten mit neovaskulärer altersbedingter Makuladegeneration. Ophthalmologe. 2016;113(3):230–9. 10.1007/s00347-015-0135-2. 26438434

[23] Morycz RK. Caregiving strain and the desire to institutionalize family members with Alzheimer’s disease: possible predictors and model development. Res Aging. 1985;7(3):329–61. 10.1177/0164027585007003002. 4059641

[24] McCaskill GM, Burgio LD, DeCoster J, Roff LL. The Use of Morycz’s Desire-to-Institutionalize Scale Across Three Racial/Ethnic Groups. J Aging Health. 2011;23(1):195–202. 10.1177/0898264310381275 20847361

[25] Prosiegel M, Böttger S, Schenk T, König N, Marolf M, Vaney C. Der erweiterte Barthel-Index (EBI) - eine neue Skala zur Erfassung von Fähigkeitsstörungen bei neurologischen Patienten [The extended Barthel Index (EBI): a new scale to assess disability in neurological patients]. Neurologie und Rehabilitation. 1996;2:7–13.

[26] Dooley WK, Shaffer DR, Lance CE, Williamson GM. Informal care can be better than adequate: Development and evaluation of the Exemplary Care Scale. Rehabilitation Psychology. 2007;52(4):359–69. 10.1037/0090-5550.52.4.359

[27] McClendon MJ, Smyth KA. Quality of informal care for persons with dementia: dimensions and correlates. Aging Ment Health. 2013;17(8):1003–15. 10.1080/13607863.2013.805400. 24171485

[28] Hinrichsen GA, Niederehe G. Dementia management strategies and adjustment of family members of older patients. Gerontologist. 1994;34(1):95–102. 10.1093/geront/34.1.95 8150316

[29] Brähler E, Scheer JW. Der Gießener Beschwerdebogen (GBB) [Giessen Subjective Complaints List]; Manual. 3rd ed. Bern: Huber; 1983.

[30] Weis MLD, Wallner M, Köck‐Hódi S, Hildebrandt C, McCormack B, Mayer H. German translation, cultural adaptation and testing of the Person‐centred Practice Inventory – Staff (PCPI‐S). Nurs Open. 2020;7(5):1400–11. 10.1002/nop2.511. PMC7424447 32802360

[31] White DL, Newton-Curtis L, Lyons KS. Development and Initial Testing of a Measure of Person-Directed Care. Gerontologist. 2008;48(suppl 1):114–23. doi/10.1093/geront/48.Supplement_1.114. 18694992

[32] Löwe B, Wahl I, Rose M, Spitzer C, Glaesmer H, Wingenfeld K, et al. A 4-item measure of depression and anxiety: validation and standardization of the Patient Health Questionnaire-4 (PHQ-4) in the general population. J Affect Disord. 2010;122(1–2):86–95. 10.1016/j.jad.2009.06.019. 19616305

[33] Tarlow BJ, Wisniewski SR, Belle SH, Rubert M, Ory MG, Gallagher-Thompson D. Positive Aspects of Caregiving: Contributions of the REACH Project to the Development of New Measures for Alzheimer’s Caregiving. Res Aging. 2004;26(4):429–53. doi/10.1177/0164027504264493

[34] Schulz S, Töpfer N, Hillebrand M, Pfeiffer K, Wilz G. Development and psychometric evaluation of brief scales for assessing two dimensions of psychosocial resource utilization in family caregivers. GeroPsych. 2024;37(4):199–211. 10.1024/1662-9647/a000337.

[35] Pendergrass A, Beische D, Becker C, Hautzinger M, Pfeiffer K. An abbreviated German version of the Sense of Competence Questionnaire among informal caregivers of relatives who had a stroke: development and validation. Eur J Ageing. 2015;12(3):203–13. 10.1007/s10433-015-0342-3. PMC5549239 28804355

[36] Hughes ME, Waite LJ, Hawkley LC, Cacioppo JT. A Short Scale for Measuring Loneliness in Large Surveys: Results From Two Population-Based Studies. Res Aging. 2004;26(6):655–72. 10.1177/0164027504268574 PMC2394670 18504506

[37] Hautzinger M, Bailer M, Hofmeister D, Keller F. Allgemeine Depressionsskala. 2nd ed. Göttingen: Hogrefe; 2012.

[38] Beierlein C, Kovaleva A, László Z, Kemper CJ, Rammstedt B. Kurzskala zur Erfassung der Allgemeinen Lebenszufriedenheit (L-1). Zusammenstellung sozialwissenschaftlicher Items und Skalen (ZIS). 2015; Available from: http://zis.gesis.org/Id/zis229. Accessed 1 Sep 2025.

[39] Given CW, Given B, Stommel M, Collins C, King S, Franklin S. The Caregiver Reaction Assessment (CRA) for Caregivers to Persons with Chronic Physical and Mental Impairments. Res Nurs Health. 1992;15:271–83. 10.1002/nur.4770150406 1386680

[40] Moore GF, Audrey S, Barker M, Bond L, Bonell C, Hardeman W, et al. Process evaluation of complex interventions: Medical Research Council guidance. BMJ. 2015;350:h1258. 10.1136/bmj.h1258. PMC4366184 25791983

[41] Lent RW, Hill CE, Hoffman MA. Development and validation of the Counselor Activity Self-Efficacy Scales. J Couns Psychol. 2003;50(1):97–108. 10.1037/0022-0167.50.1.97.

[42] Hahn D, Weck F, Witthöft M, Kühne F. Assessment of Counseling Self-Efficacy: Validation of the German Counselor Activity Self-Efficacy Scales-Revised. Front Psychol. 2021;12:780088. 10.3389/fpsyg.2021.780088/full PMC8716828 34975668

[43] Faul F, Erdfelder E, Lang AG, Buchner A. G*Power 3: a flexible statistical power analysis program for the social, behavioral, and biomedical sciences. Behav Res Methods. 2007;39(2):175–91. 10.3758/bf03193146 17695343

[44] Borm GF, Fransen J, Lemmens WAJG. A simple sample size formula for analysis of covariance in randomized clinical trials. J Clin Epidemiol. 2007;60(12):1234–8. 10.1016/j.jclinepi.2007.02.006. 17998077

[45] Cooper B, Bickel H, Schäufele M. The ability of general practitioners to detect dementia and cognitive impairment in their elderly patients: a study in Mannheim. Int J Geriatr Psychiatry. 1992;7(8):591–8. 10.1002/gps.930070809.

[46] R Core Team. R: A Language and Environment for Statistical Computing [Internet]. Vienna: R Foundation for Statistical Computing; 2025. Available from: https://www.r-project.org.

[47] SAS Institute Inc., Cary, NC, USA, 2025.

[48] IBM Corporation, SPSS Statistics for Windows (Version 31.0), Armonk, NY, 2025.

[49] Badr H, Acitelli LK. Re-thinking dyadic coping in the context of chronic illness. Curr Opin Psychol. 2017;13:44–8. 10.1016/j.copsyc.2016.03.001. 28813292

[50] Carparelli C, Oyebode JR, Riley GA. The association between the quality of care provided by family members and the quality of their relationship with the care receiver in dementia: a mixed-methods observational study. Inquiry. 2025;62:00469580241307798. 10.1177/00469580241307798. PMC11773529 39873140

